# Prevalence and characterization of gastrointestinal and ectoparasites in long-tailed macaques (*Macaca fascicularis*) from ecotourism regions of Aceh, Indonesia

**DOI:** 10.14202/vetworld.2025.1527-1539

**Published:** 2025-06-15

**Authors:** Muhammad Hanafiah, Teuku Reza Ferasyi, Erdiansyah Rahmi, Winaruddin Winaruddin, Kartika Dewi, Roliamy Saputri, Nisrima Redukmi

**Affiliations:** 1 Laboratory of Parasitology, Faculty of Veterinary Medicine, Universitas Syiah Kuala, Banda Aceh, Indonesia; 2 Laboratory Veterinary Public Health of Universitas Syiah Kuala, Banda Aceh, Indonesia; 3 Laboratory of Histology and Embryology, Faculty of Veterinary Medicine, Universitas Syiah Kuala, Banda Aceh, Indonesia; 4 Museum Zoologicum Bogoriense, Research Center for Biosystematics and Evolution, National Research and Innovation Agency (BRIN), Cibinong, Indonesia; 5 Veterinary Education Study Program, Faculty of Veterinary Medicine, Universitas Syiah Kuala, Banda Aceh, Indonesia

**Keywords:** Aceh, ecotourism, gastrointestinal parasites, *Macaca fascicularis*, *Oesophagostomum bifurcum*, scanning electron microscopy, *Trichuris trichiura*, zoonosis

## Abstract

**Background and Aim:**

Long-tailed macaques (*Macaca fascicularis*) serve as critical sentinels for zoonotic disease surveillance due to their ecological proximity to human populations. Understanding their parasitic burden is vital for conservation and public health, particularly in ecotourism areas where human-primate interactions are frequent. This study aimed to investigate the prevalence, diversity, and morphological characteristics of gastrointestinal (GI) and ectoparasites in *M. fascicularis* across four natural habitats in Aceh Province, Indonesia.

**Materials and Methods:**

A total of 100 fecal samples were collected from wild macaques at four sites: Pulau Weh Sabang Nature Tourism Park, Kuala Langsa Mangrove Forest, Saree (Aceh Besar), and Aceh Jaya. The parasitological examination involved the centrifugation method, lactophenol staining, and scanning electron microscopy (SEM). Parasites were identified based on egg morphology and adult worm anatomy.

**Results:**

Of the 100 samples analyzed, 45% tested positive for GI parasites. Nematode prevalence was highest (80%), followed by protozoa (10%) and ectoparasites (10%). Identified nematodes included *Ancylostoma* spp. (70%), *Oesophagostomum* spp. (50%), *Strongyloides* spp. (40%), *Ascaris* spp. (30%), *Enterobius* spp. (20%), and *Trichuris* spp. (10%). Protozoan (*Balantidium* spp.) and ectoparasitic (*Psoroptes* spp.) infections were less common. Mixed infections were more frequent (70%) than single infections (30%). Adult worms examined through SEM and lactophenol staining were confirmed to be two nematode species: *Oesophagostomum* (*Conoweberia*) *bifurcum* and *Trichuris trichiura*, both with zoonotic potential.

**Conclusion:**

The high prevalence of nematodes, particularly zoonotic species, underscores the importance of monitoring parasitic infections in *M. fascicularis* residing in tourist-exposed areas. This study highlights the utility of combining traditional and advanced diagnostic techniques to enhance parasite surveillance. Integrating health assessments of wild primate populations into conservation programs is recommended to mitigate zoonotic risk and support One Health objectives.

## INTRODUCTION

Primates, members of the class *Mammalia*, encompass approximately 264 known monkey species globally [1]. Long-tailed macaques are a significant attraction for both domestic and international tourists. According to the Department of Forestry, Pulau Weh Sabang is located at an elevation of approximately 28 meters above sea level (m asl), whereas the Kuala Langsa Mangrove Forest lies at around 25 m asl [2]. Individuals of *Macaca fascicularis* residing in Pulau Weh Sabang are characterized by a darker or more blackish hue compared to those found in other regions. On a global scale, more than 350 primate species have been identified, with *M. fascicularis* categorized within the Order *Primates*. Habitat destruction has played a major role in the substantial decline of primate populations worldwide, as highlighted by Falah and Sabri [3].

The occurrence of long-tailed macaques in ecotourism destinations is closely connected to their ecological influence on the surrounding environments. It is imperative to conduct further studies focusing on infectious agents, particularly parasites, to identify potential risk factors involved in the transmission of zoonotic diseases to humans. Anthropogenic disturbances, such as deforestation interfere with host-parasite interactions among primate populations [4]. Wild primates are of particular interest due to their historical association with the origin of zoonotic diseases, such as malaria [5]. Numerous studies have documented the presence of gastrointestinal (GI) parasites in monkeys. Adhikari *et al*. [6] found a high prevalence of GI parasite infections in macaques from Kathmandu, Nepal, where 87.6% of fecal samples tested positive. Among helminthic parasites, *Trichuris* spp. and *Strongyloides* spp. had the highest prevalence rates, each accounting for 31.13%, followed by hookworms (24.52%) and strongyle-type eggs (23.58%). Rahmi *et al*. [7] documented parasitic infections in macaques on Weh Island, Sabang, identifying protozoa *Eimeria* spp. (12%) and *Strongyloides* spp. (8%). The prevalence of GI parasites among wild *M. fascicularis* in the Pulau Weh Sabang ecotourism region remains relatively low. Based on the aforementioned data and concerns, the present study aims to determine whether wild long-tailed macaques inhabiting the Taman Wisata Alam (TWA) Weh Sabang Island, Kuala Langsa Mangrove Forest Area, Saree Aceh Besar, and Aceh Jaya are infected with specific types of helminths.

Although various studies have investigated GI parasitic infections in non-human primates (NHPs), including *M. fascicularis*, there remains a paucity of comprehensive data on the parasitic burden of wild populations inhabiting ecotourism zones in Aceh Province, Indonesia. Previous research has largely focused on either captive primate populations or those residing in limited geographic areas, often using basic parasitological techniques without morphological confirmation at the species level. Moreover, the application of advanced diagnostic methods, such as scanning electron microscopy (SEM), in combination with classical techniques, such as lactophenol staining and centrifugation, has rarely been employed in integrated field-based assessments. This limits the taxonomic resolution and zoonotic risk assessment of parasitic infections in macaques that frequently interact with human populations. Furthermore, there is inadequate understanding of how different environmental contexts, including forest type and anthropogenic disturbance, influence the prevalence and diversity of helminthic and protozoan infections in these primates. These gaps constrain the development of effective health monitoring frameworks and zoonotic disease prevention strategies within the One Health paradigm.

This study aims to fill the existing knowledge gap by conducting a comprehensive parasitological survey of wild *M. fascicularis* populations across four ecotourism and conservation sites in Aceh Province, Indonesia: Pulau Weh Sabang Nature Tourism Park, Kuala Langsa Mangrove Forest Area, Saree (Aceh Besar), and Aceh Jaya. Specifically, the study seeks to (i) estimate the prevalence and diversity of GI and ectoparasites through fecal analysis using the centrifugation technique, (ii) identify adult nematodes based on morphological features using lactophenol staining and SEM, and (iii) assess the potential public health implications of the detected parasites due to their zoonotic relevance. By integrating classical parasitological techniques with high-resolution imaging tools, this investigation provides critical insights for primate conservation and zoonosis prevention in human-wildlife interface zones. The findings are expected to inform evidence-based management practices that align with the objectives of biodiversity conservation and the One Health approach.

## MATERIALS AND METHODS

### Ethical approval

All experimental procedures involving animals were approved by the Aceh Natural Resources Conse-rvation Center, Directorate of Natural Resources Con-servation, Ministry of Environment (Approval No. SK.66/K.20/TU/KSA.2.2/08/2024).

### Study period and location

**Figure 1:**
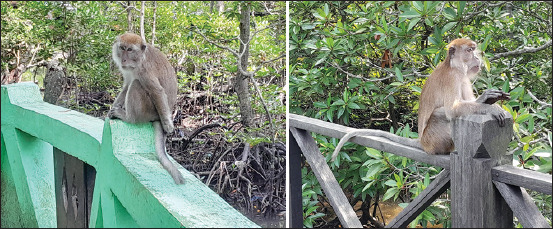
*Macaca fascicularis* individuals from which fecal samples were collected.

**Figure 2:**
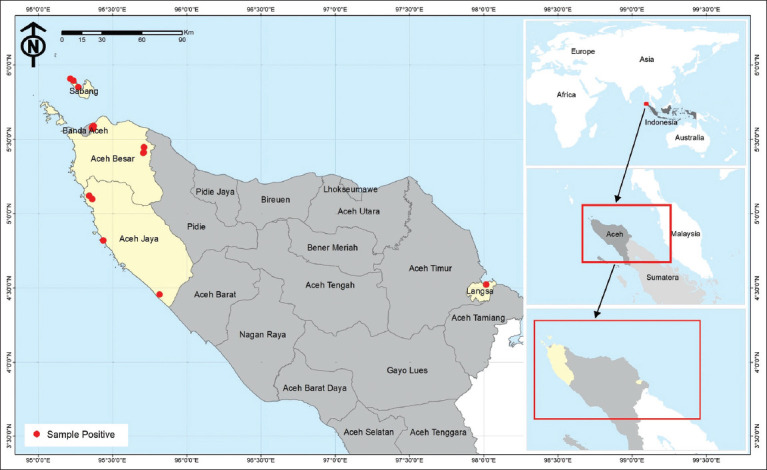
Location of *fascicularis* feces sampling [Source : Aceh Qanun Number 19 of 2013 concerning the Aceh Regional Spatial Planning (RTRW) for 2013-2033].

This study was conducted between June and October 2023 across four habitats of long-tailed macaques in Aceh Province, namely, Weh Sabang Island Nature Tourism Park (TWA), Kuala Langsa Man-grove Forest Area, Saree (Aceh Besar), and Aceh Jaya. Sampling locations and corresponding macaque popu-lations are depicted in Figures 1 and 2. All sample processing was carried out at the Research Center for Biosystematics and Evolution, National Research and Innovation Agency.

### Sample collection

Fecal samples were collected from 100 long-tailed macaques, with 25 samples were randomly obtained from each of the four study sites TWA, Kuala Langsa Mangrove Forest Area, Saree (Aceh Besar), and Aceh Jaya.

Sample size was determined using the formula [8]:

n = 4PQ / L²

Where,: n = minimum sample size required, P = estimated proportion of events (prevalence), Q = 1 - P (proportion of those who did not experience the event) and L = absolute error limit (desired margin of error)

The fresh feces were collected immediately after *M. fascicularis* defecated with soft feces criteria and placed in a sterile defecation container, stored in insulated boxes containing ice packs, and transported to the Parasitology Laboratory at Universitas Syiah Kuala. Samples were maintained at 4°C and processed within 24 h. Direct identification of helminths was performed shortly after the sample was acquired.

### Fecal processing

#### Formalin-ethyl acetate concentration technique (FECT)

Fecal parasite concentration was determined using the FECT following the protocol described by Smith *et al*. [9]. Two grams of feces were dissolved in 15 mL of 0.85% normal saline and vortexed thoroughly. The suspension was filtered through wet gauze into a 15 mL centrifuge tube. Samples were centrifuged at 500 × *g* for 5 min using a Thermo Scientific Heraeus Multifuge X3R centrifuge. After discarding the supernatant, 7 mL of 10% formalin was added to the pellet. Following homogenization and 5 min of settling at room temperature (25°C or 77°F), 3 mL of ethyl acetate was introduced and mixed. The mixture was centrifuged again at 500 × *g* for 5 min, and the supernatant was discarded. The remaining sediment was homogenized using a cytopipette. A drop of this sediment was stained with 1% Lugol’s iodine solution (Merck, Germany) and examined microscopically at 100× and 400× magnifications using an Olympus BX53 light microscope (Olympus Corporation, Tokyo, Japan) equipped with a DP74 camera [10].

### Parasite identification

#### Centrifuge method

Two grams of feces were homogenized with distilled water and centrifuged at ~2795 × *g* for 5 min. The supernatant was discarded, and the remaining pellet was mixed with saturated sodium chloride solution. After a second centrifugation, a smear was prepared from the upper layer and examined under 100 × magnification to detect parasitic structures [11].

#### Light microscopy of adult worms

Identification of nematodes was conducted using light microscopy based on morphological features, following the classification criteria described by Hasegawa *et al*. [10]. Adult worms obtained from the GI tract were fixed in AFA solution (93 parts 70% ethanol, 5 parts formaldehyde, and 2 parts glacial acetic acid). Fixed specimens were rinsed with saline, cleared in lactophenol, and examined under an Olympus BX50 compound microscope. Imaging was performed using an Olympus DP22 camera mounted on the microscope. Morphological and morphometric characteristics were further evaluated using a LEICA DM2500 microscope equipped with an image capture system, in accordance with the methods described by Thwaite [12] and Pinheiro *et al*. [13].

#### SEM

Nematode identification was performed using SEM, following the criteria established by Hasegawa *et al*. [9]. Parasites were fixed in 2.5% glutaraldehyde (Merck, Germany), dehydrated through a graded ethanol series (50% to 100%), and vacuum-dried using an aspirator (Iuchi Seieido Co., Osaka, Japan). Each specimen was mounted on an aluminum stub and gold-coated at 5–8 mA for 5 min using an IB-2 ion coater (Eiko Co., Tokyo, Japan). SEM observation was conducted at an accelerating voltage of 5 kV using a JEOL IT-200 Scanning Electron Microscope (JEOL Ltd., Japan), and representative images were captured to document morphological differences among worm specimens [12].

## RESULTS

### Parasite identification

#### Overview

Identification of nematodes was conducted using both light microscopy and SEM. The *M. fascicularis* individuals examined, and their respective locations are presented in Figures 1 and 2.

#### Identification using centrifuge method

A total of 100 fecal samples collected from *M. fascicularis* across four ecotourism sites – TWA, Kuala Langsa Mangrove Forest Area, Saree (Aceh Besar), and Aceh Jaya – were analyzed using the centrifugation technique. Among these, 45 samples (45%) tested positive for GI parasites (Table 1). The prevalence of GI nematode infections at each site is summarized in Table 1.

**Table 1:** Prevalence rate of gastrointestinal parasites in *Macaca fascicularis*.

No.	Sample origin	Positive	Negative	Total prevalence (%)
1.	Weh Sabang Island Nature Tourism Park (TWA)	15	10	60
2.	Kuala Langsa Mangrove Forest Area, Aceh Indonesia	15	10	60
3.	Saree Aceh Besar	8	17	32
4.	Aceh Jaya	7	18	28
	Total	45	55	45

Across the study locations, six distinct GI nematodes, one protozoan, and one ectoparasite species were identified:

- (A) *Ascaris* spp.
- (B) *Enterobius* spp.
- (C) *Trichuris* spp.
- (D) *Oesophagostomum* spp.
- (E) *Strongyloides* spp.
- (F) *Ancylostoma* spp.
- (G) Protozoan: *Balantidium* spp.
- (H) Ectoparasite: *Psoroptes* spp.

**Figure 3:**
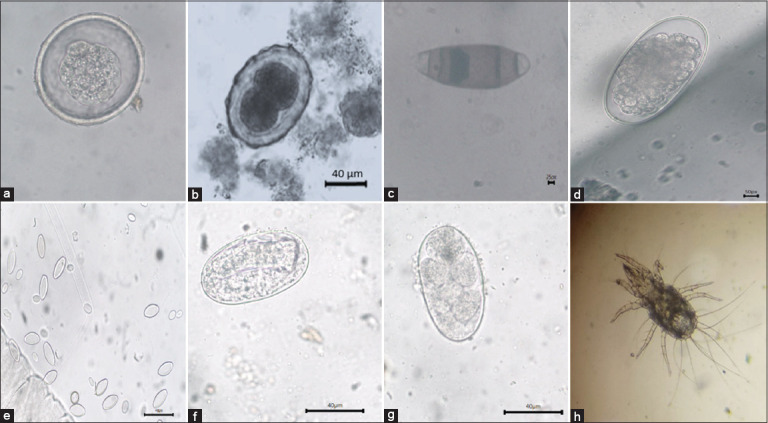
Various types of nematode eggs, protozoa, and ectoparasites identified in fecal samples of *fascicularis*. (a) *Balantidium* spp., (b) *Ascaris* spp., (c) *Trichuris* spp., (d) *Oesophagustomum* spp., (e) *Enterobius* spp., (f) *Strongyloides* spp., (g) *Ancylostoma* spp., and (h) *Psoroptes* spp. mites.

Among the nematodes, *Ancylostoma* spp. showed the highest prevalence (70%), followed by *Oesophagostomum*, *Strongyloides*, *Ascaris*, *Enterobius*, and *Trichuris* spp. (Table 2 and Figure 3).

**Table 2:** Prevalence rates of gastrointestinal parasites categorized by parasite group in Long-tailed Monkeys in the Weh Island Nature Tourism Park (TWA) Sabang, Kuala Langsa Mangrove Forest Area, Saree Aceh Besar, and Aceh Jaya (N = 100).

Parasite	Prevalence (%)
Protozoa	10
Nematoda	80
Ectoparasite	10

The values in brackets are the total number of individuals in the sample

#### Identification of adult worms

**Figure 4:**
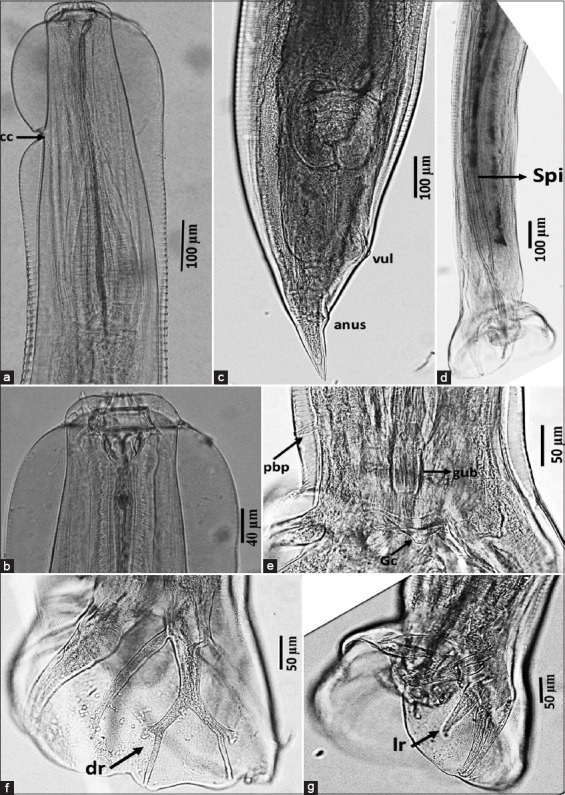
*Oesophagostomum* (*Conoweberia*) *bifurcum*. (a) Anterior part, (b) cephalic portion, (c) posterior part of female, (d) posterior part of the male showing equal and similar spicules, (e) prebursal papilla, and (f and g) bursa ray (f: dorsal ray, g: lateral ray). cc=Cervical colar, dr=Dorsal ray, gub=Gubernaculum, lr=Lateral ray, pbp=Prebursa papilla, spi=Spicula, vul=Vulva.

**Figure 5:**
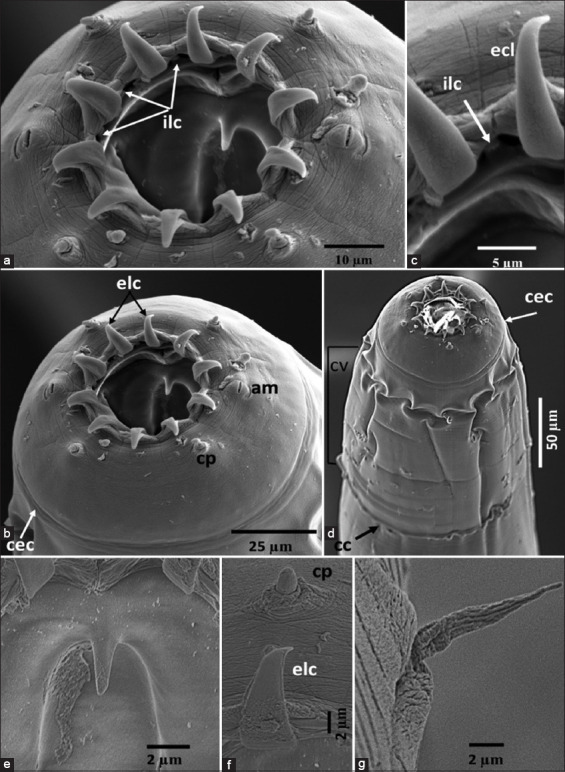
Scanning electron microscopy of *Oesophagostomum* (*Conoweberia*) *bifurcum*. (a and b) Cephalic end, (c) internal and external leaf crown, (d) anterior part, (e) buccal tooth, (f) external corona radiata leaf and cephalic papilla, and (e) cervical papilla, and (g) cervical papilla. am=Amphid, cec=Cephalic solar, cp=Cephalic papilla, cc=Cervical colar, cv=Cervical vesicle, ecl=External leaf crown elements, ilc=External leaf crown elements.

**Figure 6:**
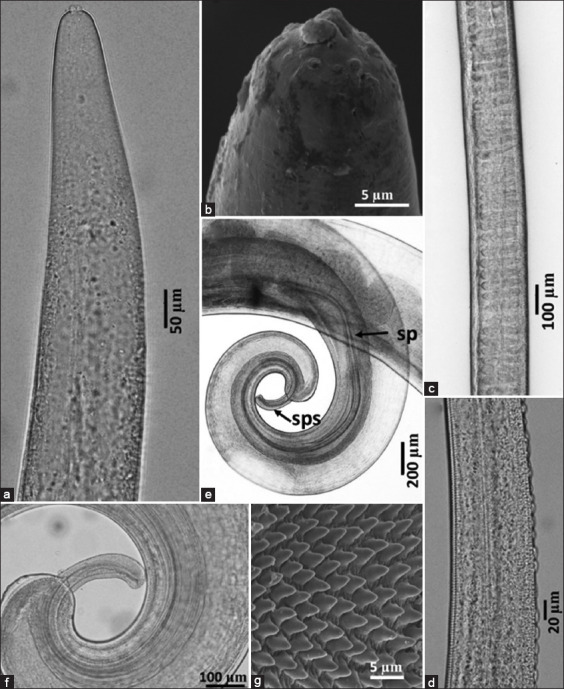
*Trichuris trichiura*. (a) Anterior part, (b) cephalic portion, (c) middle portion of stichosome, (d) bacillary band, (e) posterior part of male showing equal and similar spicules, (f) posterior part of male showing spicule sheath, and (g) detail of the spiny spicular sheath. sp=Spicule, sps=Spicule sheath.

Adult nematodes were examined using light microscopy and SEM. Two species were conclusively identified: *Oesophagostomum* (*Conoweberia*) *bifurcum* (Figures 4 and 5) and Trichuris trichiura (Figure 6).

#### O. (C.) bifurcum

This species is characterized as small-sized with a single lateral cervical collar (Figure 4a) and a prominent cervical collar (Figures 5a, b, and d). External leaf crown elements were long, tapered, and consisted of ten structures (Figures 5a, b, and c), surrounded by a pair of amphid and blunt cephalic papillae, which are four in number (Figure 5f). The buccal capsule was small and cylindrical (Figure 4b) and equipped with a buccal tooth (Figure 5e), while the cervical papillae were long and slender, positioned anterior to the esophageal swelling (Figure 5g). The excretory pore was located at the cervical groove level, and the esophagus was simple and club-shaped.

- Male (n = 1): Total length: 11.7 mm; midbody width: 360 μm; pharynx: 58 × 51 μm; esophagus length: 544 μm; posterior esophagus width: 135 μm; deirid: 479 μm; and excretory pore: 275 μm from the anterior end. Bursa was elliptical and symmetrical. Spicules were equal, slender, and measured 1.36 mm in length (Figure 4d); gubernaculum: 86 μm (Figure 4e). The dorsal ray was broad at origin with bifurcated terminal branches and a short lateral stem (Figure 4f). Ventral rays ended near the lateral lobe border (Figure 4g). Genital cone was simple, and prebursal papillae were present (Figure 4e).
- Female (n = 2): Total length: 14.38–15.44 mm; midbody width: 553–586 μm; pharynx: 58–60 × 52–54 μm; esophagus: 655–664 μm long, 125–130 μm wide. Deirid and excretory pore were located 508–512 μm and 286–317 μm from the anterior end, respectively. The vulva was positioned 408–433 μm from the posterior end. The ovejector was J-shaped, and the tail was tapering, 215–223 μm long (Figure 4c).

The morphological and morphometric data matched the description of *O. (C.) bifurcum* as outlined by Blotkamp *et al*. [14], including body lengths, buccal capsule features, external crown elements, and spicule structure. This species is commonly found in Old World monkeys, including macaques. Synonyms include *Oesophagostomum brumpti* and *Oesophagostomum* *apiostomum* [15].

#### T. trichiura

Belonging to the Order Trichocephalida (Class Enoplea), *T. trichiura*, or whipworm, is a known parasite of both humans and NHPs. In this study, only male specimens were recovered.

The specimens were consistent with the morph-ological and morphometric features reported by Rivero *et al*. [16]. The stylet protruded from the median portion of the oral cavity (Figures 6a and b), and a broad bacillary band with characteristic cuticular thickenings was present in the anterior body region (Figure 6d). Males measured 42.7–45.6 mm in total length and had a maximum thickness of 0.65–0.9 mm. The esophageal portion constituted approximately three-fifths of the total body length. The anterior body was slender and tapered, widening posteriorly. Stichosome nuclei were numerous and distinct (Figure 6c). Spicules were equal in length, measuring 2.8–3.0 mm (Figure 6e). The spicule sheath exhibited variability – cylindrical or distally expanded – when extruded and was fully covered with fine spines (Figures 6f and g).

## DISCUSSION

### Prevalence of GI nematodes

The prevalence of GI nematodes in *M. fascicularis* was 60% in macaques from the Alam Wisata Alam (TWA) Pulau Weh Sabang and Saree, Aceh Besar regions. In contrast, the prevalence in the Kuala Langsa Mangrove Forest Area and Aceh Jaya was 32% and 28%, respectively (Table 1). Variations in the types of detected GI endoparasites are likely attributable to differing habitat conditions (Figure 1). Dib *et al*. [17] reported a prevalence of 33.1% for GI parasites in NHPs in Brazil. Choong *et al*. [18] reported that the highest prevalence of GI parasites was observed among long-tailed macaques in Sulawesi (42%–73%), Malaysia (52%), and Kupang, Indonesia (86%). The prevalence of parasites is considered high when rates range between 62% and 96% [19]. Nunn and Altizer [20] found a relationship between high parasite prevalence in long-tailed macaques with their social behavior and group density.

### Comparison with other habitats

Nafisah *et al*. [21] reported lower prevalence rates of GI nematodes in *M. fascicularis* from the Wonorejo and Gunung Anyar mangrove forests, with rates of 26.67% (12/45) and 22.2% (10/45), respectively. The habitat conditions of *M. fascicularis* in Wonorejo involve close contact with humans, while in Gunung Anyar, the monkeys live primarily in trees. This observation may also apply to the present study, in which differences in GI nematode prevalence were found in fecal samples of *M. fascicularis*. Albery *et al*. [22] and Siracusa *et al*. [23] noted that GI parasitism in monkeys is influenced by several factors, including diet, age, population density, and exposure to infected individuals in the same environment.

### Infection severity and contributing factors

Elevated parasite prevalence does not necessarily correlate with visible clinical symptoms. Individuals are diagnosed with a parasitic infection when eggs or cysts are detected in their feces. Severe infections caused by worms and protozoa can reduce host populations by impairing normal physiological function [24]. Patterns of parasitic infection are shaped by factors, such as foraging behavior, grooming, social interactions, age, sex, geographic distribution, and transmission strategies [23].

Furthermore, differences in GI parasite prevalence may result from insufficient anthelmintic administration and inadequate hygiene practices in animals [25]. However, the current study did not collect data on anthelmintic use in macaques from TWA Weh Island, Saree Aceh Besar, Kuala Langsa Mangrove Forest, and Aceh Jaya. Therefore, the variation in prevalence may be attributed to differences in hygiene conditions in food collection areas.

Anthropogenic disturbances can significantly disrupt ecological balances, influencing parasite transmission dynamics. Transmission may occur through close contact, high group density, and elevated stress levels. Most infected individuals do not show prominent clinical symptoms before death. However, some symptoms, such as mild diarrhea, may be observable in the consistency and texture of collected feces [26].

### Role of behavior and environment in transmission

In addition to the previously mentioned factors, Brown *et al*. [27] emphasized that behavioral and environmental variables influence parasite transmission among *M. fascicularis*, including roaming behavior and interaction with conspecifics or animals from other groups. Social behaviors, including affiliative and agonistic interactions, elevate the risk of GI parasite transmission between individuals. Besides social behavior, additional contributors to parasitic infections include foraging behavior, age, sex, population density, and geographical setting.

Malaivijitnond *et al*. [28] noted that feces can facilitate the spread of helminth infections by contaminating feed, water, and soil. Animal behavior, particularly soil contact, can also contribute to worm transmission. Infection prevalence among macaques varies by species, with M. fascicularis exhibiting the highest positivity rates compared to M. nemestrina and M. maura, possibly due to its larger population size. According to Malla *et al*. [29], poor environmental conditions and large animal populations can enhance helminth transmission among healthy individuals, lead-ing to elevated prevalence.

Bryanta and Hallema [30] observed that helminth transmission is more efficient in tropical climates due to high humidity. Warm, moist environments promote the survival of worm larvae. For example, Surabaya Zoo administers ivermectin every 3 months as a preventive measure against helminth infections, which is effective against several parasite types.

### Parasite diversity and comparative data

This study identified eight parasites, namely, *Enterobius*, *Ancylostoma*, *Trichuris*, *Strongyloides*, *Asc-aris*, and *Oesophagostomum*, Protozoan infections involved *Balantidium*, while mite infestations were represented by *Psoroptes* spp (Table 3). In contrast, Kurniawati *et al*. [31] reported four nematode and three protozoan species in *M. fascicularis* from Baluran National Park. Identified species included *Trichostrongylus*, *Strongyloides*, *Trichuris*, *Enterobius*, *Entamoeba*, *Blastocystis*, and *Giardia*. Zanzani *et al*. [32] identified seven protozoa – *Entamoeba coli*, *Endolimax nana*, *Iodamoeba bütschlii*, *Chilomastix mesnili*, *Balantidium coli*, and two nematode species: *Trichuris* and *Oesophagostomum* in captive *M. fascicularis*.

**Table 3:** Prevalence of each gastrointestinal parasite in Long-tailed Monkeys in the Weh Island Nature Tourism Park (TWA) Sabang, Kuala Langsa Mangrove Forest Area, Saree Aceh Besar, and Aceh Jaya (N = 100).

Parasite	Prevalence (%)
*Trichuris* spp.	10
*Ancylostoma* spp.	70
*Oesophagostomum* spp.	50
*Strongyloides* spp.	40
*Enterobius* spp.	20
*Ascaris* spp.	30
Mite	10
*Balantidium* spp.	10

The values in brackets are the total number of individuals in the sample

Zoonotic endoparasites are among the most common diseases shared between humans and NHPs [33]. Transmission of parasites between primates and humans is frequent. Taylor *et al*. [34] reported that 75% of pathogenic endoparasites in humans are of zoonotic origin. Common protozoan parasites shared by humans and NHPs include *Entamoeba*, *Giardia*, *Iodamoeba*, and *Chilomastix* [35, 36], while helminths include *Ascaris*, *Strongyloides*, *Ancylostoma*, *Trichuris*, *Oesophagostomum*, *Enterobius*, and *Hymenolepis* [36].

### Soil conditions and transmission risk

Data from Table 2 show that nematode prevalence exceeded that of protozoan and ectoparasitic infections. This is likely due to moist, shaded soil conditions favorable for the development of infective nematode stages. Dwipayanti *et al*. [37] suggested that intestinal nematode transmission in monkeys is facilitated by behaviors involving the ingestion of soil-contaminated food. The frequency of nematode transmission is linked to fecal soil contamination and monkey activity levels, temperature, and environmental conditions [13]. The social lifestyle of *M. fascicularis*, which live in large groups, contributes to elevated prevalence rates of intestinal nematodes [4].

This study identified two infection types: Single infections (30%) and double infections (70%) (Table 4). These results differ from those of Dwipayanti *et al*. [37], who reported a 77.8% rate of mixed infections and 17.8% of single infections. High rates of mixed infections may be due to the social behavior of *M. fascicularis*, which live in groups of 6–100 individuals [38], increasing opportunities for contact and transmission.

**Table 4:** Gastrointestinal parasite infection status among *M. fascicularis* in the Weh Island Nature Tourism Park (TWA) Sabang, Kuala Langsa Mangrove Forest Area, Saree Aceh Besar, and Aceh Jaya (N = 100).

Parasite	Prevalence (%)
Single infection	30
Multiple infection	70

The values in brackets are the total number of individuals in the sample

### Helminth and protozoan distribution

When comparing helminth and protozoan prevalence, Pourrut *et al*. [39] concluded that higher helminth rates in *M. fascicularis* may result from cum-ulative exposure in adult animals. The relatively low protozoan prevalence may be linked to the use of formalin as a fixative. Li *et al*. [40] reported protozoan infections at 40.1% and helminth infections at 29.6%. In contrast, Adhikari and Dhakal [41] reported helminth and protozoan prevalence of 52.68% and 40.86%, respectively, in hanuman langurs and rhesus monkeys. Zang *et al*. [42] observed higher helminth (93.23%) than protozoan (89.96%) infection rates in rhesus monkeys.

### Morphological confirmation: *Oesophagostomum bifurcum*

Adult worm identification using lactophenol and SEM confirmed the presence of *O. (C.) bifurcum* (Figures 4 and 5) and *T. trichiura* (Figure 6), which are localized in the cecum and large intestine of various monkey species. These findings are consistent with Zanzani *et al*. [32], who reported *Trichuris* and *Oesophagostomum* with a low prevalence and egg-per-gram value of 2.03%.

Lactophenol-stained morphological examination of *O. bifurcum* male worms from *M. fascicularis* revealed features, such as the anterior and cephalic ends, the female’s posterior, and the male bursa copulatrix (Figure 4). SEM analysis showed 10 external leaf crown elements, internal and external leaf crowns, teeth, cervical papillae, and other diagnostic structures (Figure 5). This supports Blotkamp *et al*. [14], who reported 10–12 leaf crowns in *O. bifurcum*. According to Krepel *et al*. [43], infestation with *O. bifurcum* results in nodule-like abscesses, hence the name “nodular worms.”

*Oesophagostomum aculeatum* is limited to Asia and infects primates, such as Japanese macaques and *M. fascicularis*, while *O. bifurcum* and *Oesophago-stomum* *stephanostomum* infect African primates and Asian rhesus monkeys (*Macaca* *mulatta*) [44]. *O. bifurcum* can infect both humans and NHPs and causes significant disease due to larval encystment in the intestinal wall [45]. Although NHPs have been suggested as potential reservoirs, differences in distribution between humans and NHPs in Ghana sug-gest cryptic species or variants [46, 47]. De Gruijter *et al*. [48] concluded that *O. bifurcum* in humans and NHPs follows different transmission patterns, suggesting a limited public health risk from cross-infection.

### Morphological confirmation of *T. trichiura*

Lactophenol staining of male *T. trichiura* worms from *M. fascicularis* revealed a longer anterior region, the posterior end with spicules, a distinct mouth and stylet, and tuberculate bands (Figure 6). SEM images showed cephalic structures, similar spicules, spicule sheaths, and spiny features on the spicule sheath (Figure 6). Emikpe *et al*. [49] confirmed *T. trichiura* infection in *M. fascicularis* and other primates [50], and it is recognized as a highly pathogenic species.

*T. trichiura* exhibits a filamentous anterior and broader posterior region. The anterior section has two cuticular patterns – transverse grooves on the left side and tuberculate bands on the right [51]. Under higher magnification, raised circular bodies are visible. The anterior tapers toward the head, showing a longitudinal bacillary band (Figure 6b). The mouth is surrounded by two concentric circles of papillae and contains a central stylet (Figure 6a). The posterior end is curved ventrally (Figure 6d).

*Trichuris* spp. are commonly found in wild NHPs, such as colobus monkeys, macaques, baboons, and chimpanzees. This study recorded a 17% prevalence of Trichuris. Previous reports indicated 10% in Kandi Wildlife Park (West Sumatra), 8.82% on Tinjil Island, and 21% in *M. bullata* from rural Bangladesh. In Asian NHPs, prevalence ranges up to 30%. A molecular study by Kurniawati *et al*. [31] has shown that some *Trichuris* species are host-specific, while others may be zoonotic due to their genetic proximity to human strains.

Eggs of *T. trichiura* are lemon-shaped, measure 25.69 μm, and have bipolar plugs (Figure 1c). The eggs are easily identified through flotation and are commonly found in both humans and primates. Infec-tive eggs are ingested through contaminated food. Clinically, infections may be asymptomatic or present with symptoms, such as colitis, mucus, and blood in the stools. Severe infections can lead to rectal prolapse due to intestinal irritation and muscle weakness (musculus levator ani) [52].

## CONCLUSION

This study revealed a notable prevalence of GI and ectoparasites in wild *M. fascicularis* populations inhabiting ecotourism zones in Aceh Province, Indonesia. The overall prevalence of GI parasitism reached 60% in some locations, with *Ancylostoma* spp. (70%) and *Oesophagostomum* spp. (50%) as the most frequently detected nematodes. The morphological confirmation of *O. (Conoweberia.) bifurcum* and *T. trichiura* using light microscopy and SEM underscores the zoonotic relevance of these species and enhances the diagnostic accuracy beyond conventional methods.

From a practical standpoint, these findings underscore the importance of routine parasitological monitoring in primate ecotourism habitats. The results highlight the urgent need for integrated One Health interventions, particularly in areas where human-primate interactions are common. Public awareness campaigns, improved habitat hygiene, and targeted anthelmintic administration may serve as effective measures to mitigate the transmission of zoonotic parasites.

A key strength of this study is its combined use of fecal analysis, lactophenol staining, and SEM for parasite identification, allowing for robust morphological validation. Furthermore, the multi-site sampling appr-oach provided comprehensive geographic insights into parasite distribution across diverse habitats.

However, the study has certain limitations. First, the absence of data on host factors, such as age, sex, immune status, and nutritional condition restricted the ability to analyze host-specific risk factors. Second, the lack of molecular characte-rization limited the taxonomic resolution, especially in distinguishing cryptic or closely related parasite species. Third, the study did not assess environmental parameters (e.g., soil moisture and vegetation cover) quantitatively, which could have provided additional ecological context.

Future research should incorporate molecular tools to explore genetic diversity and zoonotic potential of the identified parasites. Longitudinal surveillance, including host health indicators and environmental sampling, is recommended to track temporal trends and inform evidence-based policy.

This study contributes critical baseline data on the parasitic burden and zoonotic risks associated with *M. fascicularis* in Aceh’s ecotourism regions. The findings underscore the need for integrated wildlife health monitoring and intersectoral collaboration to prevent the emergence and spread of zoonotic infections at the human–wildlife interface.

## DATA AVAILABILITY

All the generated data are included in the manuscript.

## AUTHORS’ CONTRIBUTIONS

MH: Designed and managed all research permits and drafted the manuscript. TRF and ER: Data analysis and drafted the manuscript. RS and NR: Collected the samples, KD: Identified adult nematode worms and performed light and electron microscopic photographs. WW: Collection of worms in the field. All authors have read and approved the final manuscript.
